# Supplementary material for: Light-induced analgesia provides a drug-free optical method for pain relief via activation of TRAAK k+ channels
Source: Nat Commun. 2026 Jan 26;17:620. doi: 10.1038/s41467-025-67819-w (PMC12834984; doi:10.1038/s41467-025-67819-w)
Supplement: Supplementary file 1 — Supplementary Information [file 41467_2025_67819_MOESM1_ESM.pdf]

Supplementary Information for

Light-Induced Analgesia Provides a Drug-Free Optical Method for Pain Relief via Activation of TRAAK K+ Channels

Marion Bied[1,2,3], Arnaud Landra-Willm[1,2,3], Anne Amandine Chassot[1,2,3], Edward Francisco Mendez-Otalvaro[4], Benjamin Sueur[5], Kilian Roßmann[6], Elvira de la Peña Garcia[7], Pascal Fossat[5], Stephen J Tucker[8,9], Jacques Noël[2,3,10], Wojciech Kopec[4,11], Felix Viana[7], Johannes Broichhagen[6], Eric Boué-Grabot[5], Guillaume Sandoz[1,2,3*]

[1]Université Côte d'Azur, CNRS, INSERM, iBV, France.

[2]Laboratories of Excellence, Ion Channel Science and Therapeutics, Nice, France.

[3]Fédération Hospitalo-Universitaire InovPain, Cote d'Azur University, University Hospital Centre Nice, Nice, France.

[4]Computational Biomolecular Dynamics Group, Max Planck Institute for Multidisciplinary Sciences, Göttingen, Germany.

[5]Université de Bordeaux, CNRS, Institut des Maladies Neurodégénératives, UMR 5293, F- 33000, Bordeaux, France.

[6]Leibniz-Forschungsinstitut für Molekulare Pharmakologie (FMP), Berlin, Germany.

[7]Instituto de Neurociencias de Alicante, Universidad Miguel Hernández-CSIC, San Juan de Alicante, Spain.

[8]Department of Physics, Clarendon Laboratory, University of Oxford, Oxford, U.K.

[9]Kavli Institute for Nanoscience Discovery, University of Oxford, Oxford, U.K.

[10]Université Côte d'Azur, CNRS, INSERM, IPMC, Valbonne, France.

[11]Department of Chemistry, Queen Mary University of London, London, U.K.

* Corresponding author, e-mail: sandoz@unice.fr

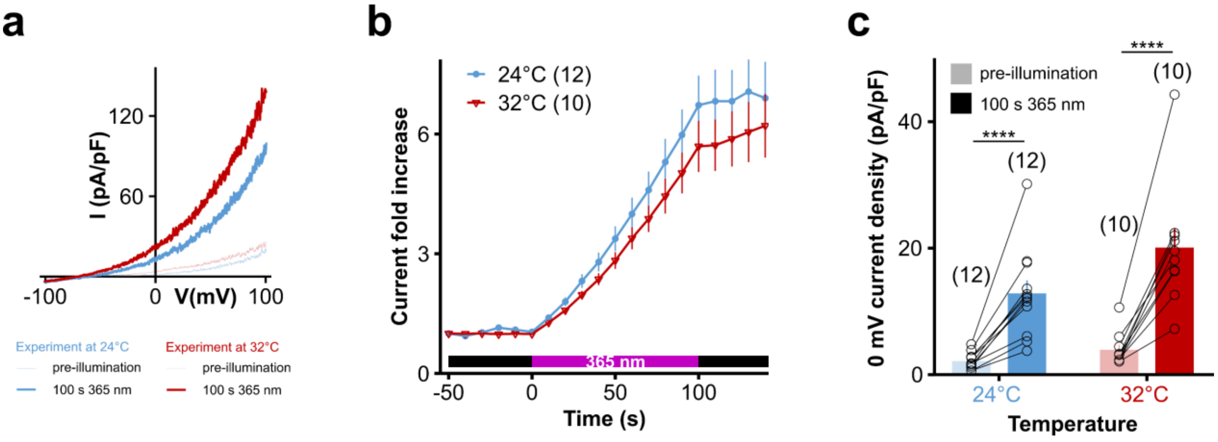

Supplementary Figure 1: **365 nm light activation of mTRAAK occurs similarly at room (24°C) and skin (32°C) temperature.**

a. Representative whole-cell current traces of HEK293T cells expressing mTRAAK recorded at 24°C or 32°C, before and after a 100 s 365 nm illumination. (Traces for one given temperature were obtained from the same cell. For each temperature condition, similar traces have been obtained independently 10 to 12 times.)

b. Time course of mTRAAK responses to a 100 s 365 nm-centred illumination recorded at 24°C or 32°C. Current fold increases were computed at 0 mV relative to the pre-illumination 0 mV current of the cell.

c. Comparison of mTRAAK 0 mV current densities at 24°C or 32°C, before and after a 100 s 365 nm-centred illumination (carried out at 24°C or 32°C accordingly). Statistical analyses compare the 0 mV current densities of mTRAAK before and after the illumination either at 24°C or 32°C using a Bonferroni-corrected paired t-test (*** p<0.0001).

Data shown are mean ± s.e.m. The number of recorded cells is indicated in the figure. Source data are provided in the Source Data file.

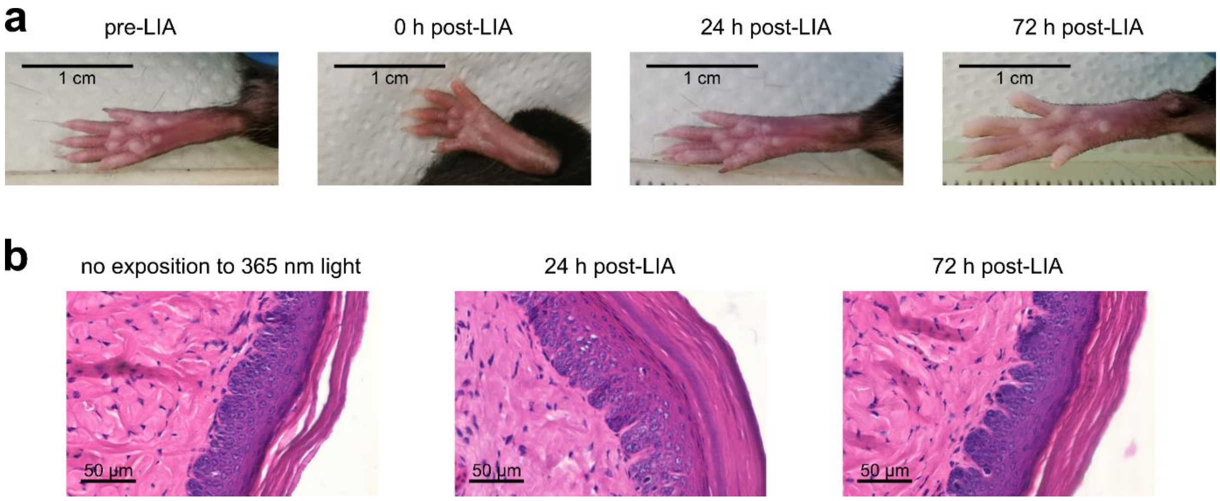

Supplementary Figure 2: **LIA treatment does not produce inflammatory side effect.**

a. Pictures of mice paws before, after, 24 or 72 hours after the end of LIA treatment, showing the absence of effect of LIA (10 min 365 nm illumination) on mice skin. Paws originate from different mice.

b. Representative shematoxylin-eosin-stained sections of hindpaw skin that did not undergo LIA, or that was sampled 24 or 72 hours after the end of the LIA treatment. No apparent sign of inflammation is visible on LIA treated skin samples.

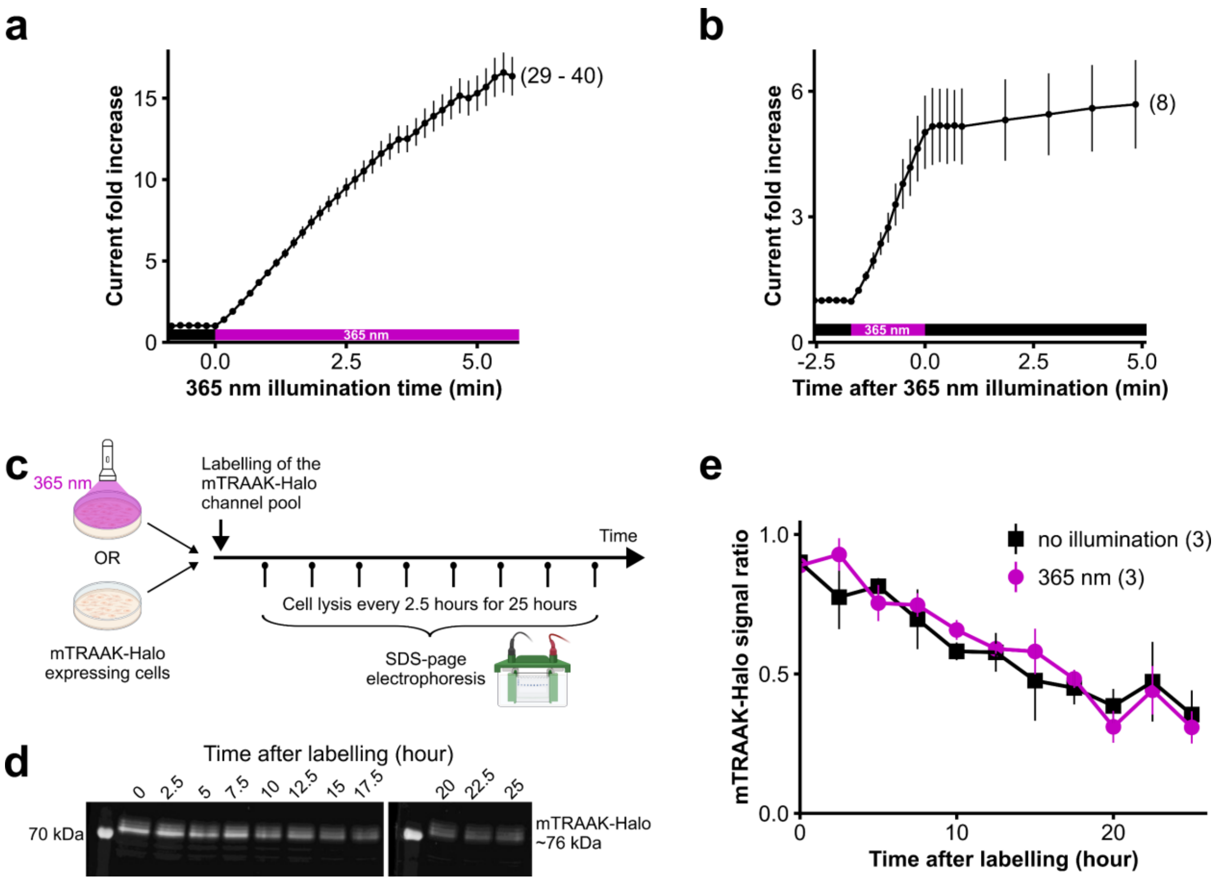

Supplementary Figure 3: **Time dependence of the 365 nm-induced mTRAAK current increase.**

a. Time course of mTRAAK current increase induced by long-lasting 365 nm illuminations. Current fold increases were computed at 0 mV relative to the pre-illumination 0 mV current.

b. Time course of mTRAAK 0 mV current fold increase following a 100 s (1 min 40 s) 365 nm illumination. Note that mTRAAK current remains stable up to 5 min following the end of the illumination.

c. Diagram explaining the experiment carried out to assess mTRAAK degradation timescale. Halo-tagged mTRAAK proteins were labelled using the Halo-TMR cell permeable ligand. Cells were then lysed every 2.5 hours for 25 hours and the resulting cell lysates were analysed through SDS page electrophoresis. The quantity of labelled Halo-tagged mTRAAK channel was quantified through the fluorescent intensity of the ~76 kDa band corresponding to the Halo-tagged mTRAAK channels. Before labelling of the Halo-tagged mTRAAK channels, half of the cell plates were illuminated with 365 nm light for 10 min to assess the impact of 365 nm illumination on the degradation timescale of mTRAAK (created in BioRender).

d. Representative SDS-page gel revealed by fluorescence imaging and containing cell lysates collected up to 25 hours after the end of the labelling of the Halo-tagged mTRAAK channel pool. The expected weight of the Halo-tagged mTRAAK channel is 76 kDa. Here cells were not illuminated with 365 nm light at the beginning of the experiment. (Similar gels have been obtained twice independently.)

e. Quantification of the remaining labelled Halo tagged mTRAAK channels over time, defined as the ratio of the fluorescent intensity of the 76 kDa band at each time point with respect to the intensity of the most fluorescent band.

Data shown are mean ± s.e.m. The number of recorded cells or experiments is indicated in the figure. Source data are provided in the Source Data file.

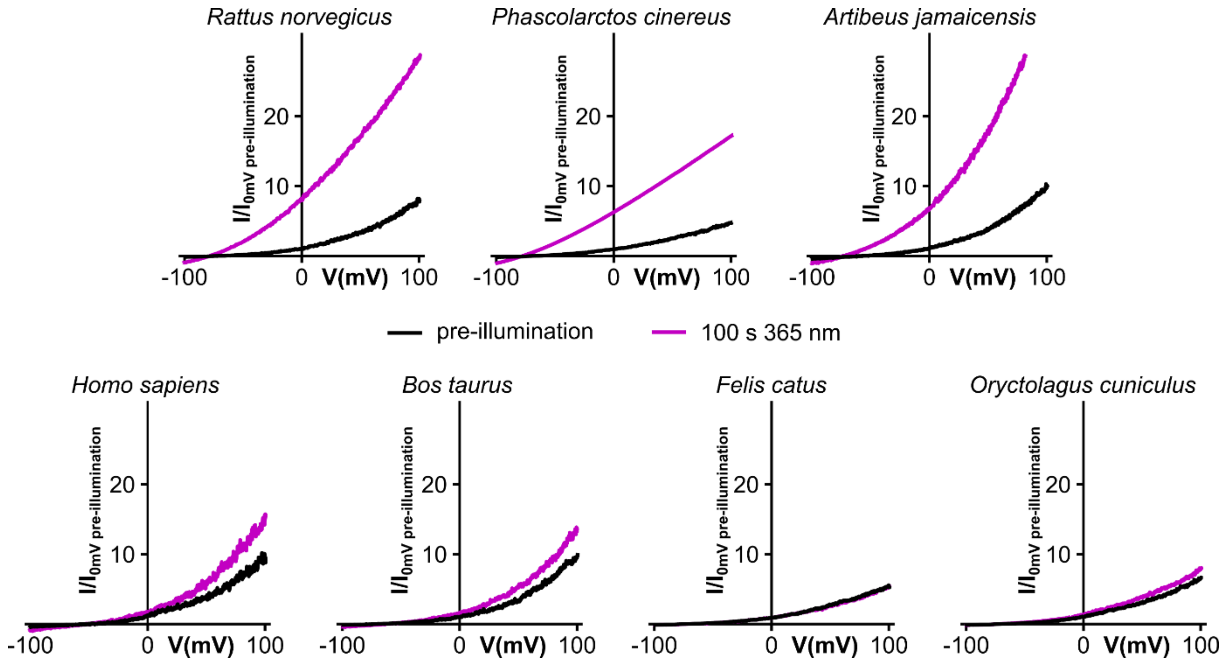

Supplementary Figure 4: **TRAAK light-sensitivity is species-dependent.**

Representative normalized whole-cell current traces of HEK293T cells expressing TRAAK mammalian orthologs before and after a 100 s UV-A365 nm illumination. Currents were elicited by voltage-clamp ramps (-100 to +100 mV, 800 ms) and were normalized by the 0 mV current of the cell recorded before illumination. Each channel has been recorded independently 5 to 12 times with similar results, as specified on figure 2i.

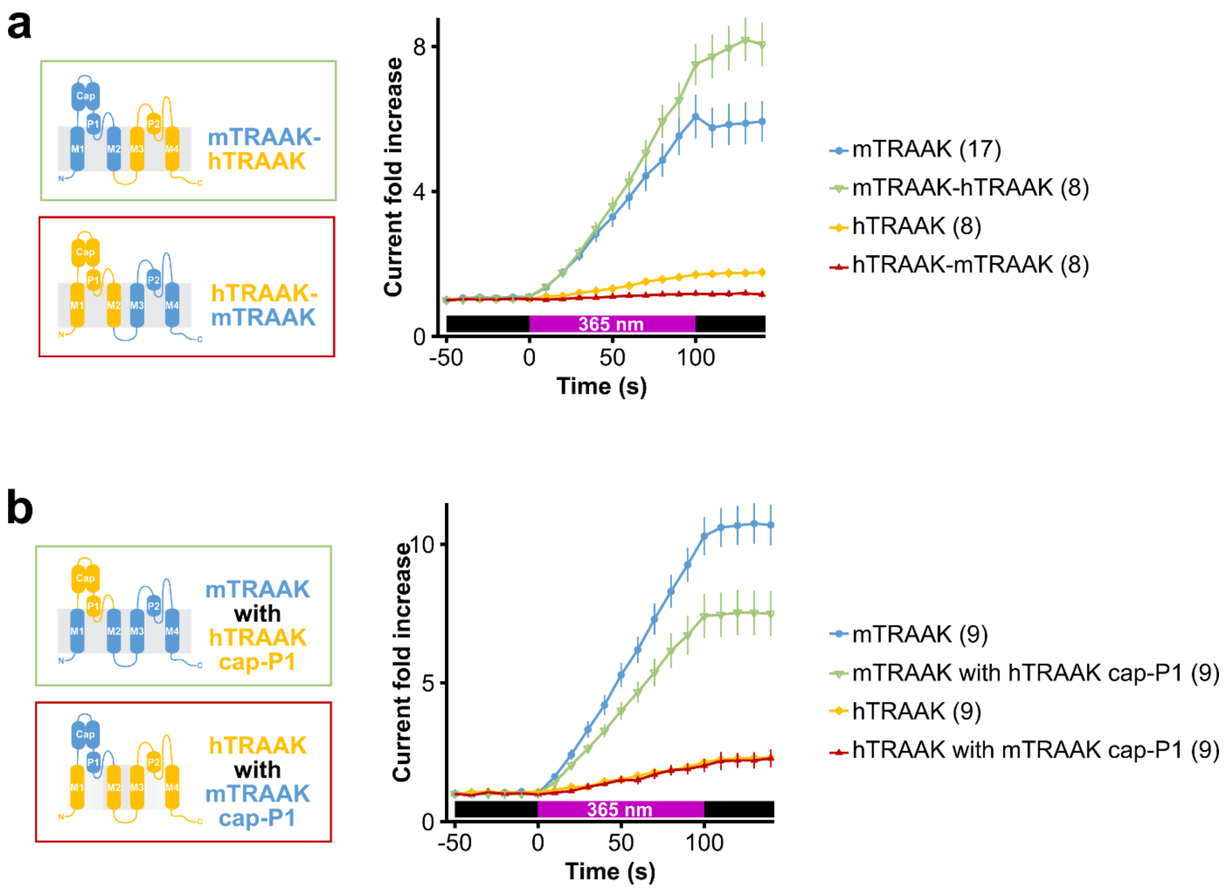

Supplementary Figure 5: **The light sensitive domain of mTRAAK lies within the first and/or second transmembrane segments.**

a. Time course of responses of human-mouse TRAAK chimeric channels to a 100 s 365 nm illumination. Current fold increases were computed at 0 mV relative to the pre-illumination 0 mV current of the cell. Cartoons on the left indicate chimeric channel composition. One can deduce that the light sensitive domain of mTRAAK lies within the first half of mTRAAK.

b. Similar to a for human-mouse TRAAK cap + P1-swapping chimeric channels, showing that the light-sensitive domain of mTRAAK is outside of the channel's extracellular cap and P1 loop.

Data shown are mean ± s.e.m. The number of recorded cells is indicated in the figure. Source data are provided in the Source Data file.

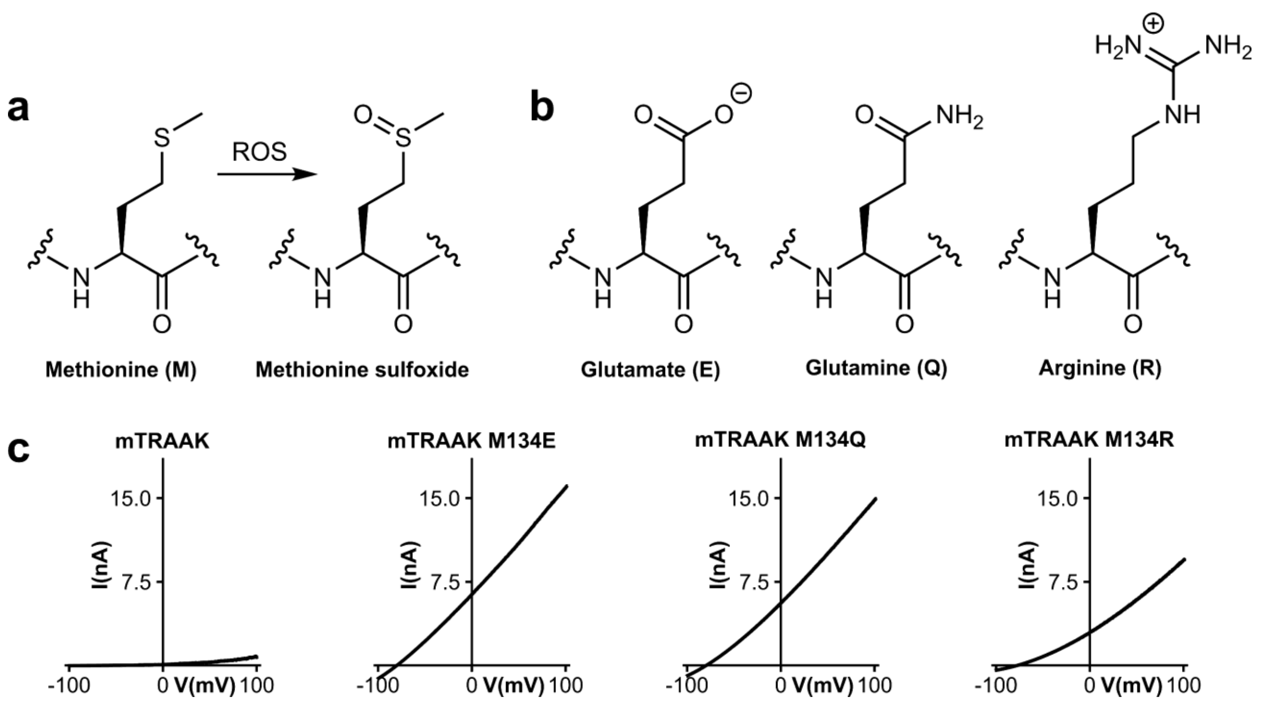

Supplementary Figure 6: **Oxidation of methionine 134 modifies the electronic density at its core, promoting the activated-state of TRAAK.**

a. Oxidation of methionine into methionine sulfoxide. Skeletal formulas of methionine and methionine sulfoxide are represented, highlighting the addition of a double-bond oxygen in methionine sulfoxide which results in a modified electronic density. (ROS : Reactive Oxygen Species).

b. Skeletal formulas of glutamate, glutamine and arginine, three polar amino acids, featuring a negative full (glutamate) or partial charge (glutamine), or a positive charge (arginine).

c. Representative whole-cell current traces of HEK293T cells expressing mTRAAK, mTRAAK M134E, mTRAAK M134Q or mTRAAK M134R. Currents were elicited by voltage-clamp ramps (-100 to +100 mV, 800 ms). Each channel has been recorded independently 8 to 23 times with similar results, as specified on figure 4e.

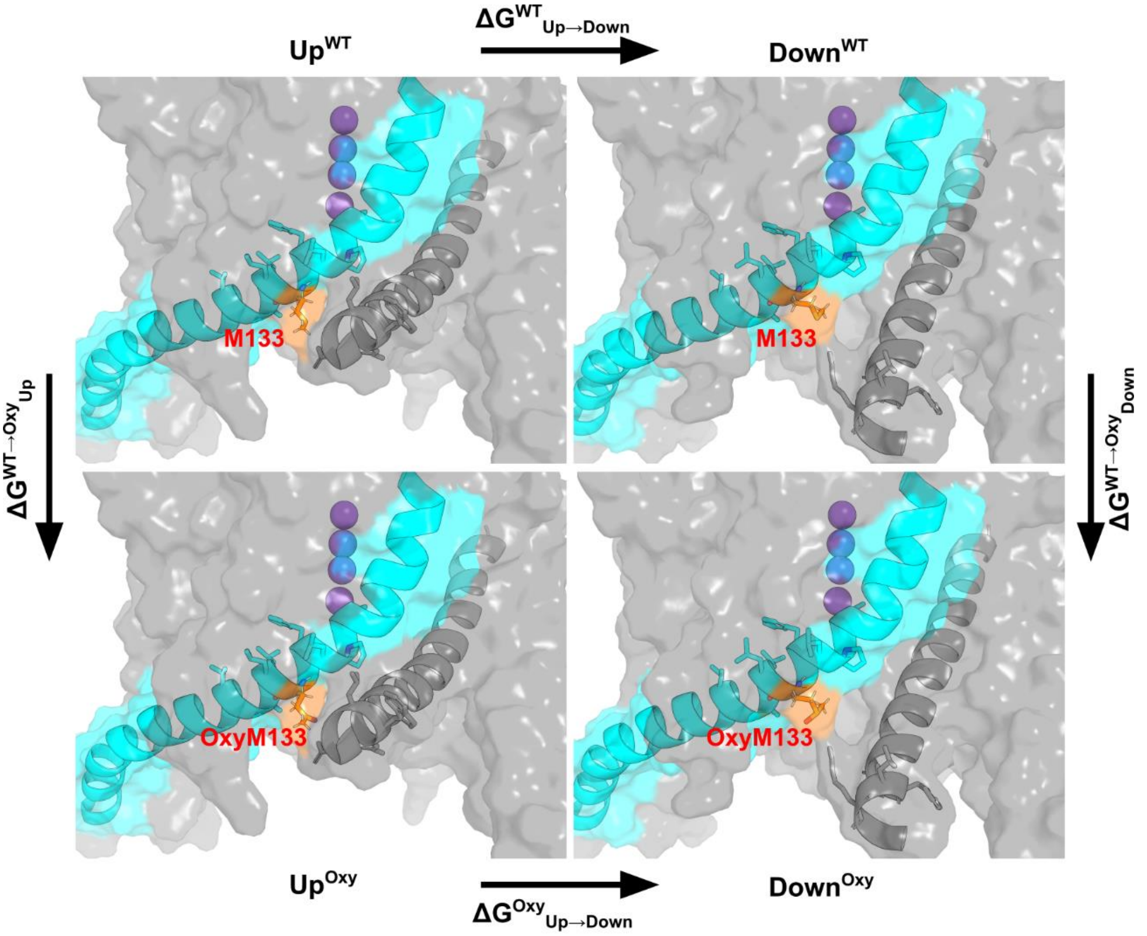

$$\Delta\Delta G^{Oxy}_{Up\rightarrow Down} = \Delta G^{Oxy}_{Up\rightarrow Down} - \Delta G^{WT}_{Up\rightarrow Down} = \Delta G^{WT\rightarrow Oxy}_{Down} - \Delta G^{WT\rightarrow Oxy}_{Up}$$

$$\Delta\Delta G^{Oxy}_{Up\rightarrow Down} = 6.65 \pm 2.80 \text{ kJ/mol}$$

Supplementary Figure 7: **M133 oxidation favours the conductive "up" state of hTRAAK I133M.**

Thermodynamic cycle used to estimate the relative free energy difference of the up-to-down transition upon the methionine 133 (M133) to methionine sulfoxide 133 (OxyM133) oxidation in hTRAAK I133M. In this cycle, horizontal free energy changes are computationally expensive to calculate, while vertical changes can be efficiently estimated using alchemical transformations. Because free energy is a state function, the path taken is irrelevant, allowing vertical free energy differences to approximate the horizontal ones. This approach provides insights into the thermodynamic stability of the up to down transition upon the M133 oxidation.

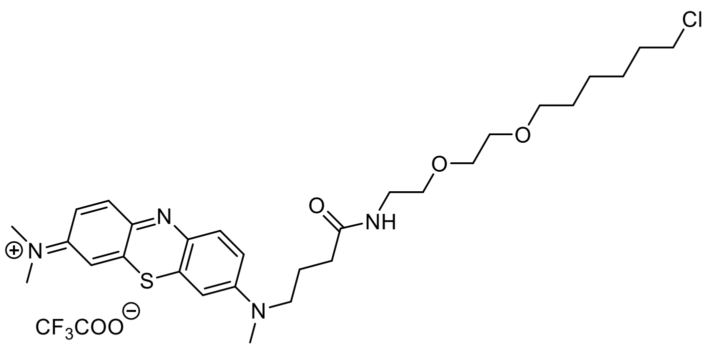

Supplementary Figure 8: **Skeletal formula of CA-ATTO-MB2** (formal name: *N*-(7-((4-((2-(2-((6-Chlorohexyl)oxy)ethoxy)ethyl)amino)-4-oxobutyl)(methyl)amino)-3*H*-phenothiazin-3-ylidene)-*N*-methylmethanaminium 2,2,2-trifluoroacetate).

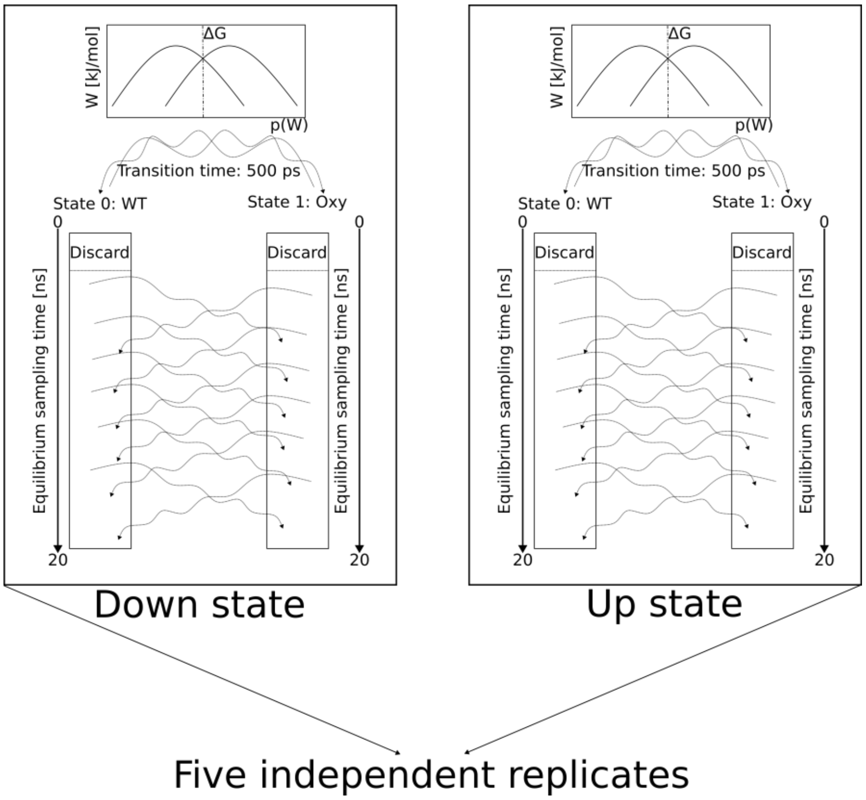

Supplementary Figure 9: **Non-equilibrium free energy calculation protocol.**

Protocol for estimating the relative free-energy change of the M133 (WT) to OxyM133 (methionine sulfoxide) transformation. Each 500 ps fast transition converts methionine into its oxidized form and vice versa. The value of the dissipated work is obtained by integrating the energy change over the transformed λ coordinate. Multiple forward and backward transitions yield work distributions, whose intersection provides the free-energy estimate via Crooks' fluctuation theorem. The procedure was performed in both UP and DOWN hTRAAK states, with the I133M mutation, across five independent replicates.

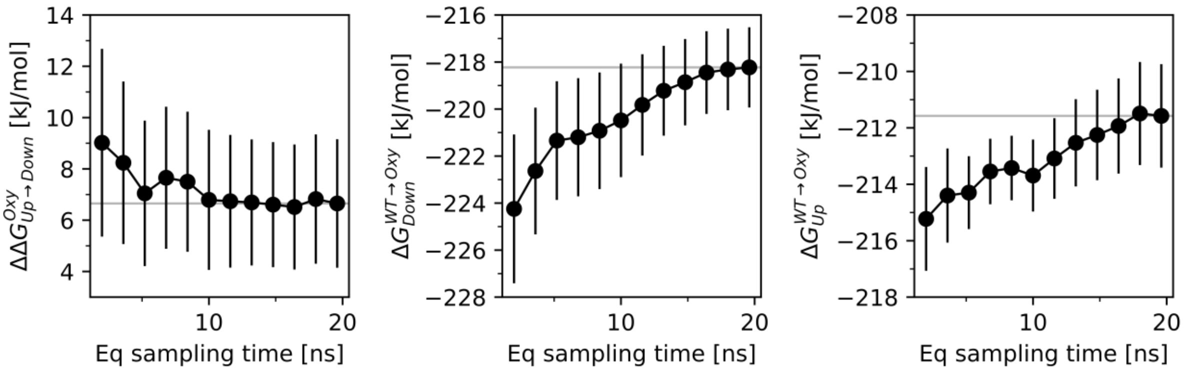

Supplementary Figure 10: **Convergence of free-energy estimates**

Convergence of free-energy estimates over the sampling time. (Left) the relative difference between the free-energy change of methionine oxidation in the DOWN state and that in the UP state; (center) the free-energy change of methionine oxidation in the DOWN state; (right) the free-energy change of methionine oxidation in the UP state. Error bars represent the standard error of the mean across five independent replicates. Eq: equilibrium.
