# Supplementary material for: Light-induced analgesia provides a drug-free optical method for pain relief via activation of TRAAK k+ channels
Source: Nat Commun. 2026 Jan 26;17:620. doi: 10.1038/s41467-025-67819-w (PMC12834984; doi:10.1038/s41467-025-67819-w)
Supplement: Supplementary file 2 — Reporting summary [file 41467_2025_67819_MOESM2_ESM.pdf]

nature portfolio

Corresponding author(s): Guillaume Sandoz

Last updated by author(s): Dec 4, 2025

Reporting Summary

Nature Portfolio wishes to improve the reproducibility of the work that we publish. This form provides structure for consistency and transparency in reporting. For further information on Nature Portfolio policies, see our Editorial Policies and the Editorial Policy Checklist.

Statistics

For all statistical analyses, confirm that the following items are present in the figure legend, table legend, main text, or Methods section.

n/a	Confirmed	
☐	☒	The exact sample size (n) for each experimental group/condition, given as a discrete number and unit of measurement
☐	☒	A statement on whether measurements were taken from distinct samples or whether the same sample was measured repeatedly
☐	☒	The statistical test(s) used AND whether they are one- or two-sided<br>*Only common tests should be described solely by name; describe more complex techniques in the Methods section.*
☒	☐	A description of all covariates tested
☐	☒	A description of any assumptions or corrections, such as tests of normality and adjustment for multiple comparisons
☐	☒	A full description of the statistical parameters including central tendency (e.g. means) or other basic estimates (e.g. regression coefficient) AND variation (e.g. standard deviation) or associated estimates of uncertainty (e.g. confidence intervals)
☐	☒	For null hypothesis testing, the test statistic (e.g. F, t, r) with confidence intervals, effect sizes, degrees of freedom and P value noted<br>*Give P values as exact values whenever suitable.*
☒	☐	For Bayesian analysis, information on the choice of priors and Markov chain Monte Carlo settings
☒	☐	For hierarchical and complex designs, identification of the appropriate level for tests and full reporting of outcomes
☒	☐	Estimates of effect sizes (e.g. Cohen's d, Pearson's r), indicating how they were calculated

Our web collection on statistics for biologists contains articles on many of the points above.

Software and code

Policy information about availability of computer code

Data collection	Pclamp11, Micro-Manager, Spike2, PhenoImagerHT 2.0.0, GROMACS 2024.2, CHARMM36m
Data analysis	Pclamp11, MSigmaPlot 11.0, GraphPad Prism 8, Excel, R 4.2.0, PhenoChart 2.0.0

For manuscripts utilizing custom algorithms or software that are central to the research but not yet described in published literature, software must be made available to editors and reviewers. We strongly encourage code deposition in a community repository (e.g. GitHub). See the Nature Portfolio guidelines for submitting code & software for further information.

Data

Policy information about availability of data

All manuscripts must include a data availability statement. This statement should provide the following information, where applicable:

- Accession codes, unique identifiers, or web links for publicly available datasets
- A description of any restrictions on data availability
- For clinical datasets or third party data, please ensure that the statement adheres to our policy

All data reported in this paper are available in the Source Data file.

Research involving human participants, their data, or biological material

Policy information about studies with human participants or human data. See also policy information about sex, gender (identity/presentation), and sexual orientation and race, ethnicity and racism.

Reporting on sex and gender	NA
Reporting on race, ethnicity, or other socially relevant groupings	NA
Population characteristics	NA
Recruitment	NA
Ethics oversight	NA

Note that full information on the approval of the study protocol must also be provided in the manuscript.

Field-specific reporting

Please select the one below that is the best fit for your research. If you are not sure, read the appropriate sections before making your selection.

☒ Life sciences ☐ Behavioural & social sciences ☐ Ecological, evolutionary & environmental sciences

For a reference copy of the document with all sections, see nature.com/documents/nr-reporting-summary-flat.pdf

Life sciences study design

All studies must disclose on these points even when the disclosure is negative.

Sample size	The sample size was based on previous experiments and homogeneity of the raw data. Sample sizes were also based on previous studies: Sandoz et al, 2012, Royal et al, 2019, Landra—Willm et al, 2023.
Data exclusions	For electrophysiology, no cells were excluded from the study unless they did not meet our recording quality standards at any time of the recording.<br>For animal experiments, no data were excluded from the analysis.
Replication	Experiments were repeated in separate cells and animals, as shown by the number of values specified in each graph.<br>Electrophysiology experiments on HEK cells were done from one cell batch, except for the experiments on mTRAAK M134I and hTRAAK I134M (Fig. 3 e-g) which were replicated twice (after validating that the outcome of the 2 experiments was the same, all values were then pooled together).
Randomization	For electrophysiology experiments, recorded cells were determined by the channel they expressed after transfection, making randomization impossible.<br>For animal experiments, the side of the hindpaw on which was applied the analgesic treatment was chosen randomly, the other side serving as a negative control. The side of the treated paw was the same for all animals used on one given experiment day.
Blinding	Data acquisition and analysis were not carried out in a blinded manner. Blinding was not possible because of the availability of researchers who carried out the experiments.

Reporting for specific materials, systems and methods

We require information from authors about some types of materials, experimental systems and methods used in many studies. Here, indicate whether each material, system or method listed is relevant to your study. If you are not sure if a list item applies to your research, read the appropriate section before selecting a response.

Materials & experimental systems

n/a	Involved in the study	
☒	☐	Antibodies
☐	☒	Eukaryotic cell lines
☒	☐	Palaeontology and archaeology
☐	☒	Animals and other organisms
☒	☐	Clinical data
☒	☐	Dual use research of concern
☒	☐	Plants

Methods

n/a	Involved in the study	
☒	☐	ChIP-seq
☒	☐	Flow cytometry
☒	☐	MRI-based neuroimaging

Eukaryotic cell lines

Policy information about cell lines and Sex and Gender in Research

Cell line source(s)	HEK cells 293T obtained from ATCC (CRL-11268) were transfected using the calcium phosphate technique to measure the current related to the transfected channels.
Authentication	The cell line has been regularly used in recent years. The cell line used was not further authenticated
Mycoplasma contamination	Cells were regularly tested for mycoplasma contamination using the MycoAlert kit from Lonza and we confirm that cells were negative for mycoplasma contamination
Commonly misidentified lines (See ICLAC register)	None.

Animals and other research organisms

Policy information about studies involving animals; ARRIVE guidelines recommended for reporting animal research, and Sex and Gender in Research

Laboratory animals	Mouse model: male and female C57BL/6J WT and double KO for TREK1 and TREK2 genes (Royal et al., 2019) adult mice (between 2.5 and 8 month old) were used for behavioral tests and ex vivo and in vivo recordings.<br>Rat model: male and female WT Wistar adult rats (aged around 3 months) were used for the behavioral test.<br>All animals were obtained from the animal facility's breeding colonies.
Wild animals	No wild animals were used in the study.
Reporting on sex	Male and female mice were used in the study. No sex-based difference was found, justifying the absence of sex-based analysis.
Field-collected samples	No field-collected samples were used in the study.
Ethics oversight	All animal experiments were conducted according to national and international guidelines and have been approved by the local ethical committees (Ministère de la Recherche, de l'Enseignement, et de l'Innovation CIEPAL - APAFIS #33381-2021093008408503 v8, APAFIS #21135-2019061914043519 v3, Ethics Committee from Universidad Miguel Hernández)

Note that full information on the approval of the study protocol must also be provided in the manuscript.

Plants

Seed stocks	NA
Novel plant genotypes	NA
Authentication	NA
